# Oil Pulling and Polyphenols: Treatment of Gingivitis Patients with ‘Itri Extra-Virgin Olive Oil’

**DOI:** 10.3390/jcm12165256

**Published:** 2023-08-12

**Authors:** Giulia Zumbo, Denise Corridore, Samantha Sciscione, Claudio Stamegna, Fabrizio Guerra, Antonella Polimeni, Iole Vozza

**Affiliations:** Department of Oral and Maxillo-Facial Sciences, Sapienza University of Rome, 00161 Rome, Italy; denise.corridore@uniroma.it (D.C.); sciscionesamantha@gmail.com (S.S.); claudio.stamegna@uniroma1.it (C.S.); fabrizio.guerra@uniroma1.it (F.G.); antonella.polimeni@uniroma1.it (A.P.); iole.vozza@uniroma1.it (I.V.)

**Keywords:** oil pulling, oral health, gingivitis, dentistry, dentistry innovations, polyphenols

## Abstract

Objectives: The aim of the study was to evaluate the effectiveness of extra virgin olive (EVO) oil and fruity oil for the treatment of gingivitis. Materials and Methods: A sample of 75 patients over 18 years of age with gingivitis induced by plaque bacteria was divided into three groups: study group A, with extra virgin olive oil; study group B, with fruity oil; and control group C. In the two study groups, EVO oil was administered as a mouthwash to patients with gingival inflammation. The protocol included a daily application of the product for 30 days, with three recalls 15 days apart. Clinical parameters of plaque formation and gingivitis, including plaque index (PI) and bleeding index (BI), were assessed at each recall and scored on a specific periodontal chart. The control group received no mouthwash treatment in addition to normal daily oral hygiene procedures, and the same clinical parameters as the study group were evaluated. Data were evaluated using SPSS 27.0 software for Windows (SPSS Inc., Chicago, IL, USA). Then, the pre- and post-treatment values of the groups were compared using Student’s *t*-test, setting *p* < 0.05 as the significance level. Results: Comparison of the three groups showed that extra-virgin olive oil was an adjuvant in the treatment of gingival inflammation, improving PI and BI. In group A, the mean plaque index showed a 48% reduction, and the bleeding index showed a 64% reduction after 30 days. In group B, the mean plaque index showed a 35% reduction and a bleeding index reduction of 43% after 30 days. Conclusions: The collected data showed significant improvements in the formation of bacterial plaque and gingivitis. The exact mechanism of such treatment is still to be elucidated. As a result of this, further studies with a different sample of patients than those used and a comparison with other products need to be addressed to verify and demonstrate the antibacterial and anti-inflammatory effects of the components of this natural product.

## 1. Introduction

Gingivitis is a reversible inflammatory pathology clinically characterized by redness, swelling, bleeding, and pain.

If left untreated, it can progress into more severe forms and lead to periodontal disease, with the loss of supporting tissues including periodontal ligaments and alveolar bone, deepening of the periodontal pocket, gingival recession, increased tooth mobility, furcation exposure, and ultimately, loss of teeth [1].

The Mediterranean diet is characterized by the high use of olive oil, vegetables, and fruits rich in antioxidants and vitamins. Adherence to a Mediterranean diet has been correlated with a lower risk of systemic and inflammatory diseases [2].

Fatty acids are increasingly recognized as a central feature of many biological processes [3]. They are essential components of phospholipids, serving as ligands for receptors in immune cells, and many of their derivatives are strong immunoregulators [4].

Lipid mediators are linked to the progression of periodontal disease due to the production of proinflammatory mediators, such as prostaglandins and leukotrienes, via the arachidonic acid cyclooxygenase/lipoxygenase pathways, such as those involving lipoxins, resolvins, and protectins, that are able to modulate the response of the host to promote resolution of inflammation and in response to periodontal pathogens. Lipoxins are natural pro-resolving molecules derived from endogenous fatty acids [5,6].

They stimulate the resolution of inflammation and promote the restoration of tissue homeostasis through several mechanisms, such as limiting the migration of polymorphonuclear neutrophils to sites of inflammation and modulating macrophage activity by inhibiting the secretion of proinflammatory cytokines [5,6].

It has been described that the release of interleukin (IL) 1 beta (IL-1B) and tumor necrosis factor alpha (TNF-A), inhibit leukocyte trafficking and attenuate the inflammatory reactions caused by *Porphyromonas gingivalis*, gives strength to the potential protective role in periodontal disease [6].

Resolvins are dietary omega-3 polyunsaturated fatty acid products (PUFA) that also stimulate the resolution of inflammation through multiple mechanisms.

Some studies have shown that resolvin E1 promotes the resolution of the inflammatory reaction, preventing a chronic response after *P. gingivalis* infection and also promoting the regeneration of periodontal connective tissue and bone [7]. The recently discovered properties of resolvins may show that these mediators may represent a new family of analgesics useful in the treatment of pain states associated with inflammation, such as arthritic and post-operative pain [8].

Many substances can be used as adjuvants for reducing the formation of plaque, but there is still little scientific evidence to validate them.

Several oil pulling studies have been done with sunflower oil, sesame oil, and coconut oil to reduce plaque-induced gingivitis. “Oil pulling” is a term that defines a traditional Ayurvedic remedy that consists of rinsing the mouth with oil [9,10,11,12]. There is still little evidence in the literature of studies concerning the properties of extra-virgin olive oil and fruity oils in relation to the oral cavity [13,14,15,16].

Extra-virgin olive oil is one of the most valuable and appreciated products of the agri-food industry, constituting an important element of the Mediterranean diet thanks to its organoleptic characteristics and its contribution to a healthy diet.

Extra-virgin olive (EVO) oil is enriched by phenolic components that are completely absent in other types of oils derived from seeds or fruits. The concentration of phenolics in virgin olive oil depends on various agronomic (genetic and geographical origin of the fruit) and technological factors. Monovarietal EVO oils show different phenolic profiles, thanks to the different abundances of secoiridoids and their derivatives. It is important to note that the process used for olive oil extraction has a major influence on the phenolic concentration. Eventually, differences in the abundance and composition of phenolics could contribute to the beneficial health properties of different monovarietal virgin olive oils by exerting different effects at the cellular level, also due to their specific levels of absorption and bioavailability. Supposedly, the multiple phenolic compounds present in virgin olive oil act synergistically or complementarily to confer benefits for the whole organism [17].

### Polyphenols in Olive Oil

Extra-virgin olive oil is rich in polyphenol molecules, especially tocopherols and biophenols, aromatic compounds, and antioxidants, which act both on the taste of the oil and on its shelf life and quality. These phenols delay the oxidation of fatty acids and the rancidity of the oil. The European Food Safety Authority (EFSA) has certified hydroxytyrosol, a polyphenol present in olives, as a substance capable of protecting blood lipids from the harmful effects of oxidative stress. The daily use of extra-virgin olive oil in the recommended doses plays an important role in human health and is recommended in a balanced diet. The phenolic compounds contained in extra-virgin olive oil, such as oleuropein and protocateuic acid, significantly increase the GPx activities [18]. The high content of monounsaturated fatty acids, in particular oleic acid, is mainly responsible for the beneficial effects of EVO oil. The main polyphenols in the oil have been found to improve the function and survival of pancreatic beta cells and improve insulin secretion, promoting glycemic control in patients with type 2 diabetes mellitus [19].

The aim of the study was to evaluate the efficacy of extra-virgin olive (EVO) oil and fruity oil for the treatment of gingivitis.

## 2. Materials and Methods 

### 2.1. Type of Study

This pilot study was conducted from March to October 2022 at the outpatient dental clinic of ASL Latina, Gaeta district. Seventy-five patients with bacterial plaque-induced gingivitis aged over 18 years were evaluated. 

The study was approved by the Institutional Local Review Board of Sapienza University of Rome in Latina (Protocol n. 2103/2022). All subjects signed an informed consent form regarding details about the nature and objectives of the study, treatment effects, and clinical conditions for inclusion before they participated in the study. The study was conducted in accordance with the Declaration of Helsinki.

### 2.2. Study Parameters

At time T0 (before nonsurgical periodontal therapy), anamnestic data were collected from individual patients to assess their readiness for and inclusion in the treatment protocol.

During the initial phase of the first visit, patients were asked various questions, focusing in particular on the patient’s health status, gender, medication intake, tobacco use, and toothbrush use and type, whether manual, electric, or both. The selected patients were well motivated, instructed in the proper use of oral hygiene tools, and made aware of the prevention and treatment of gum disease and the importance of home oral hygiene. During the professional oral hygiene session, parameters for inclusion/exclusion in the experimental study were assessed. 

### 2.3. Study Groups and Inclusion/Exclusion Criteria

After the professional oral hygiene session, patients were divided into three groups:

- Group A: Extra-virgin olive oil mouthwash;
- Group B: Fruity mouthwash;
- Group C: Control without mouthwash.

Inclusion criteria

- Male and female patients, aged over 18 years;
- Presence of at least 20 teeth in the oral cavity, excluding third molars;
- 1 < GI > 3;
- 1 < PI > 3;
- Bleeding on probing;
- Presence of pseudotasks;
- Absence of clinical attachment loss, mobility, periodontitis.

Exclusion criteria

- Poorly motivated and/or uncooperative patients;
- Patients with systemic diseases;
- Patient being treated with hormones, calcium channel blockers, cyclosporine, or phenytoin;
- Pregnant women.

### 2.4. Procedures and Protocols in the Subgroups

**Figure 1:**
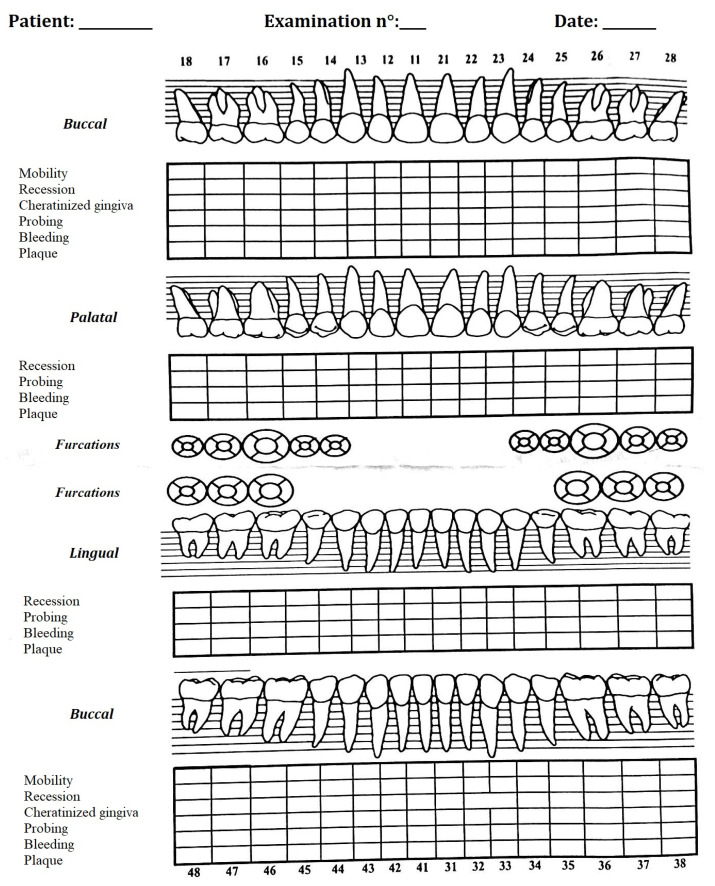
Periodontal chart used in the trial.

A periodontal chart (Figure 1) was then completed, containing gingival index, plaque, bleeding, presence of mobility, and recessions.

The gingival index used was the Silness–Löe index; a periodontal probe was used on the outer surface to determine its consistency and assess bleeding for each tooth surface element (mesial, distal, buccal, oral). The plaque index used was the O’Leary index, examining the 4 tooth surfaces (mesial, distal, buccal, oral) and reporting the presence or absence of plaque. The index was calculated by adding the number of surfaces with plaque to the number of total surfaces examined and multiplying by 100. 

The bleeding index used was the Ainamo and Bay index, which was performed by probing the 4 tooth surfaces (mesial, distal, buccal, oral) and reporting the presence or absence of bleeding. The index was calculated by summing the number of surfaces with bleeding in relation to the number of total surfaces examined and multiplying by 100. 

The presence of recessions was assessed by the Miller Index, while any dental discoloration was assessed by the dichotomous method: presence/absence followed by the mean value expressed as a percentage. Finally, gustatory perception reported by the patient was assessed.

Following completion of the chart, the patients underwent nonsurgical mechanical causal therapy. During scaling, hard and soft deposits were removed above and below the gumline using ultrasound, and teeth were polished with a toothbrush and abrasive paste. 

Candidate patients were randomly divided into three groups consisting of 25 people: two test groups and a control group. 

Patients in the test group were explained the experimental treatment to be carried out at home with the EVO oil and fruity oil mouthwash: a 5 min rinse to be performed after brushing and interproximal cleaning before going to bed for 30 days.

These patients were given 50 predosed bottles containing 10 mL of olive oil from the ‘cultivar Evo’ of the Lazio region, specifically Itrana. 

Patients in the control group were asked not to rinse with mouthwashes for 30 days while maintaining usual home oral hygiene. Recalls were performed at 15 (T1) and 30 (T2) days after the first session, and in these, the plaque index, bleeding, presence of recessions, and information such as pleasantness or non-pleasantness of taste, taste alterations, presence of dental discoloration, and cosmetic improvements noted during the course of treatment were re-evaluated.

Data analysis, plaque index, and bleeding values were performed using SPSS 27.0 software for Windows (SPSS Inc., Chicago, IL, USA). Then, the pre- and post-treatment values of the groups were compared using Student’s *t*-test, setting *p* < 0.05 as the significance level.

## 3. Results

The results of the present study are described in Table 1, which highlights the characteristics of the examined subjects who participated in the study with EVO oil and fruity oil treatment, and the subjects who participated without the use of natural mouthwash.

### 3.1. Test Group 

**Figure 2:**
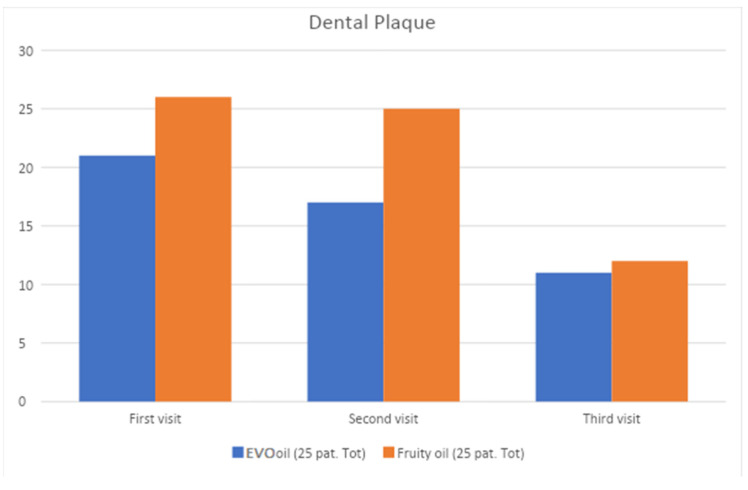
Change over time in plaque index of the test group at t0, t1, and t2.

**Figure 3:**
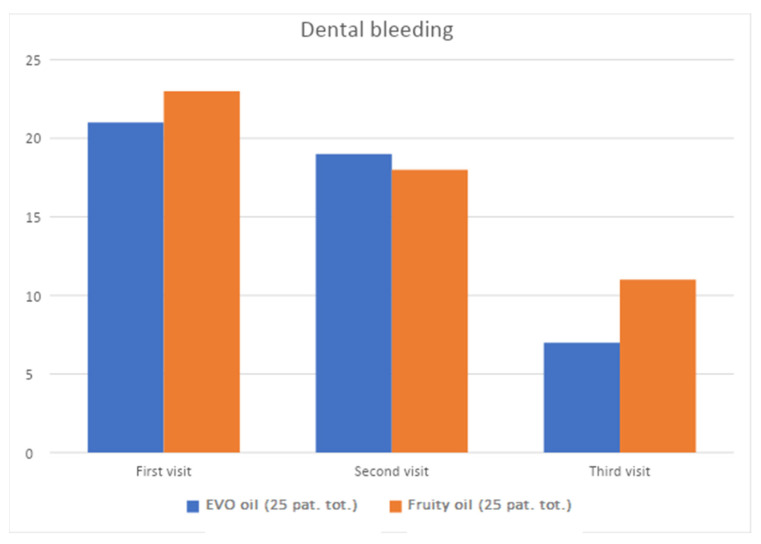
Change in bleeding index of test group at t0, t1, and t2.

At time T0 (before non-surgical periodontal therapy) and times T1 and T2 (15 and 30 days after the first session, respectively), the following data were collected: plaque index and bleeding index. The following graphs (Figure 2 and Figure 3) show the processed percentages of the collection of these data, which are intended to show the variation during therapy of the parameters.

Associated with non-surgical periodontal home treatment with home treatment with EVO oil mouthwash and fruity oil mouthwash. 

Figure 2 shows the change in plaque index of the test group over time during home treatment with oil mouthwash.

It can be seen that from t0 to t2, both test groups, experienced a significant decrease in plaque. 

Figure 3 shows the change in bleeding index over time, consequently the degree of tissue inflammation during treatment with EVO and fruity olive oils. This graph was developed by entering the bleeding indices of each patient before non-surgical mechanical therapy (t0), two weeks (t1), and four weeks (t2).

In the test group, slight variations were found for the values regarding probing depth during the recalls. These values never exceeded 3 mm in depth; therefore, they always fell within a healthy periodontal condition. No variations were found in patients with recessions. No patients in the test group presented mobility greater than a Miller index grade 0. 

None of the patients reported allergic reactions to the product.

### 3.2. Control Group

**Figure 4:**
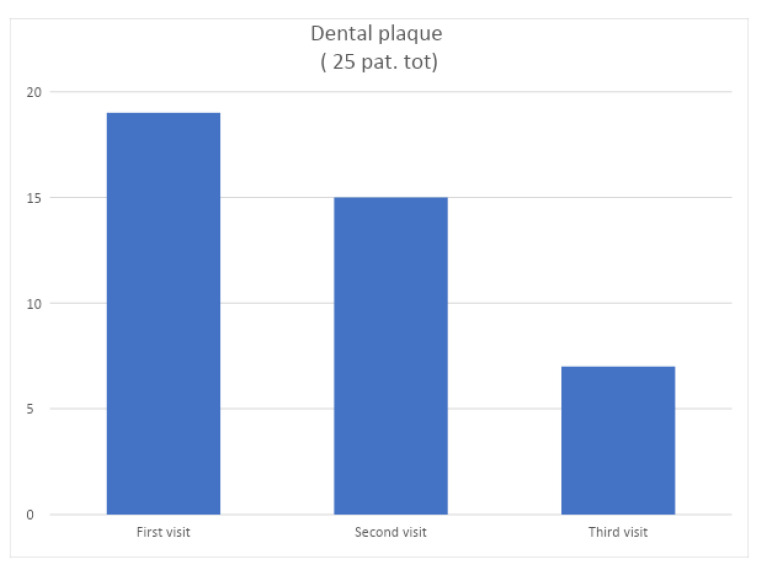
Change over time of the plaque index of the control group.

**Figure 5:**
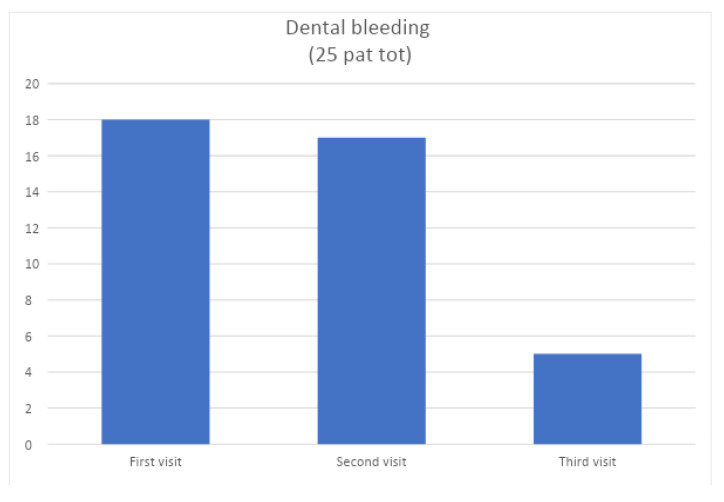
Change in bleeding indexes over time in the control group.

The following graphs (Figure 4 and Figure 5) show data collected before, during, and after non-surgical mechanical causal therapy alone and aim to show the change in these parameters over time. Recalls for the control group were made from the first session, at 15 days, and at 30 days, where the same parameters as in the test group were collected: plaque index, bleeding index, pocket depth, and presence of recessions. 

Figure 4 shows a graph with the plaque index data of the control group before non-surgical mechanical causal therapy and 30 days after it.

Figure 5 represents the bleeding index values of the control group at t0, t1, and t2.

In the control group, no changes regarding probing depth or recessions were shown during recalls. None of the patients in the control group presented a mobility index above a Miller’s index grade of 0.

### 3.3. Data Elaboration

**Figure 6:**
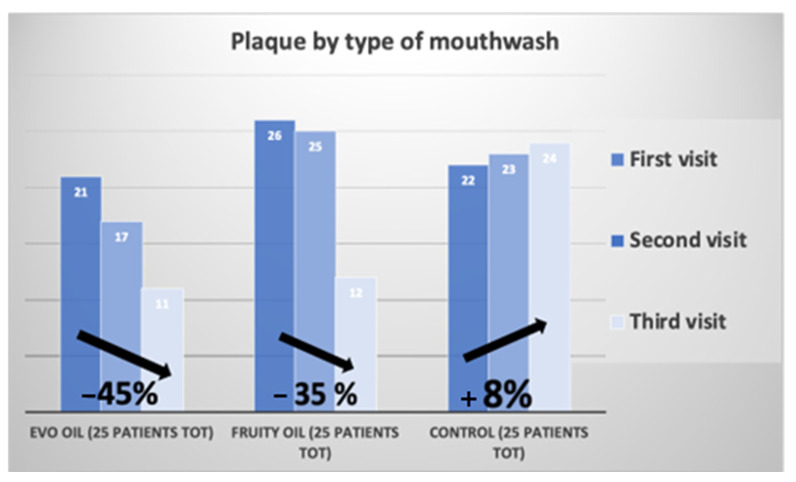
Plaque percentage over time of the test group and control group.

**Figure 7:**
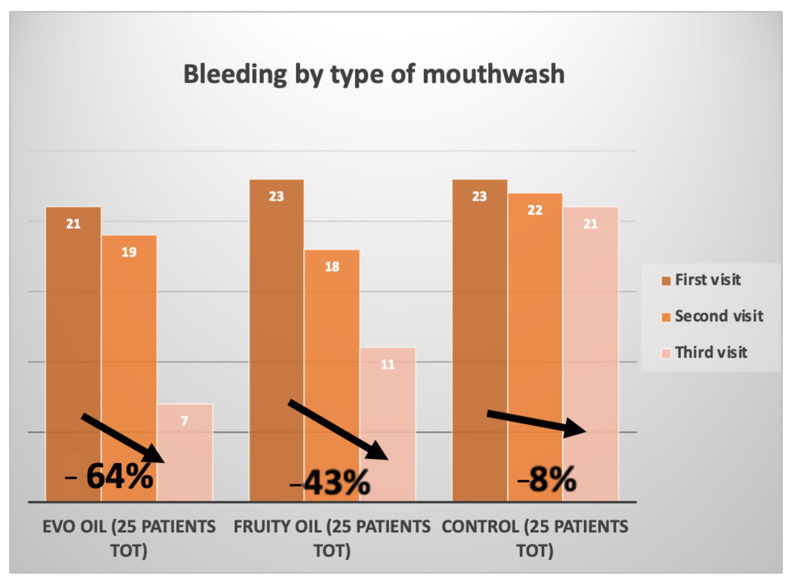
Bleeding percentage over time of the test group and control group.

Figure 6 and Figure 7 compare the plaque index and bleeding index of the two test groups and the control group. The graphs were developed by averaging the indices of the three groups at T0, before non-surgical mechanical causative treatment; T1, after 15 days; and T2, after 30 days. 

Figure 6 compares the plaque percentage, found during the three visits, of the EVO oil test group, fruity oil test group, and control group at T0, on the left; T1, in the middle; and T2, on the right.

Figure 7 compares the bleeding percentage during the three visits encountered, by the EVO oil test group, fruity oil test group, and control group at T0, on the left; T1, in the middle; and T2, on the right.

**Figure 8:**
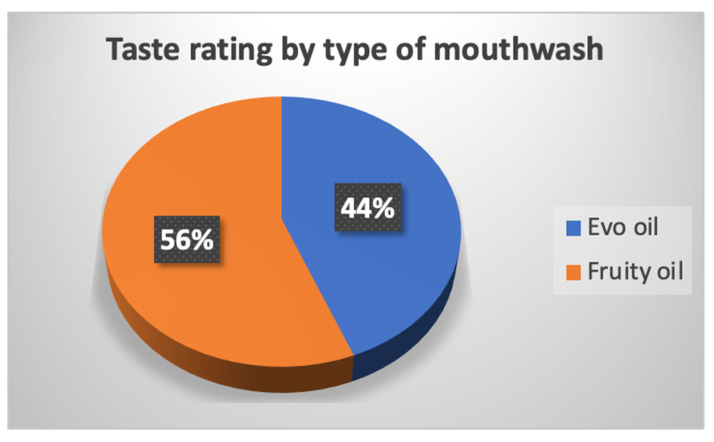
Percentage that liked the taste of the two oil-based mouthwashes.

At the end of each visit, the patients in the two test groups were asked questions about their enjoyment of the taste and whether they had noticed any improvement. Figure 8 shows the patients’ ratings during the treatments with EVO oil and fruity oil.

**Figure 9:**
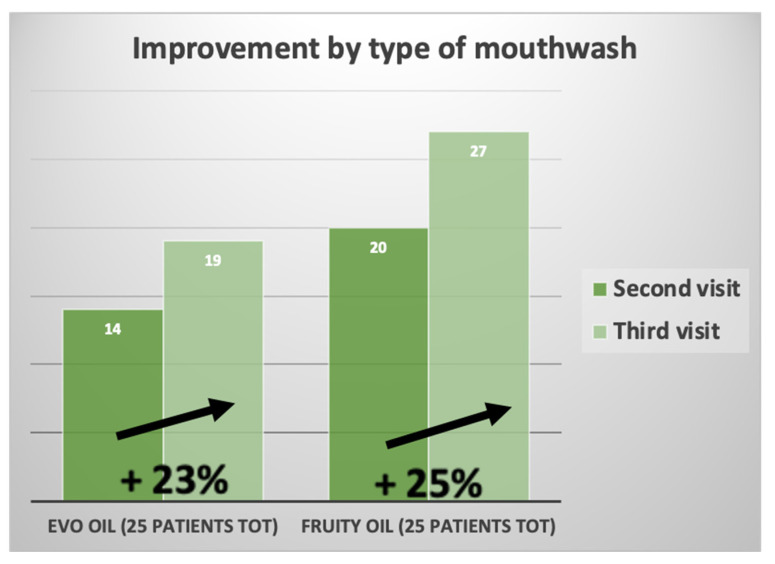
Percentage of improvement by type of mouthwash.

Figure 9 shows the data on the feeling of improvement that patients perceived during treatment with the olive oil mouthwash.

As can be seen from the graphs, patients with the fruity oil treatment liked the mouthwash more than patients with the EVO oil treatment (Figure 8). The improvement that was evaluated, as indicated by the patient’s own perception, found an improvement for both types of mouthwashes with minimal difference in fruity oil treatment. The values of plaque and bleeding indices are shown in Table 2, Table 3 and Table 4. Next, the pre- and post-treatment values of the groups were compared using Student’s *t*-test, setting *p* < 0.05 as the significance level (Table 5).

Comparing the results of the clinical data obtained following the treatments that the patients in the EVO oil group and the fruity oil group underwent with the control group, positive results can be found in both test groups, emphasizing, however, a higher percentage of those obtained than in the control group.

In the EVO oil test group, the average plaque index before non-surgical causal treatment stood at 96 percent and then decreased to an average value of 48 percent after 30 days.

In the Fruity test group, the plaque index before treatment stood at 92% and then decreased to an average value of 56% after 30 days.

In the control group, the average plaque index before treatment stood at an average value of 88% and then increased to a value of 96% after 30 days. The EVO oil test group showed a decrease in plaque index of 45%, the fruity test group showed a decrease in plaque index of 35%, and the control group showed an increase of 8% (Figure 7). The bleeding index of the EVO oil test group before non-surgical causative treatment was 96% and then decreased to a value of 32%. In the fruity oil test group, the bleeding index stood at a value of 84% and then decreased to a value of 44%, while in the control group it stood at a value of 92% and then decreased to a value of 84%. The EVO oil test group showed a decrease in bleeding index of 64%, and the fruity oil test group showed a decrease in bleeding index of 43%, while the control group showed a decrease of only 8% (Figure 8).

Comparison of the plaque and bleeding indices of the three test groups with Student’s *t*-test showed a slight difference in the averages before treatment, with a *p* value (significance level) < 0.05. The plaque index was found to be *p* = 0.008, while the bleeding index was *p* = 0.018. After 30 days, the plaque index was found to be *p* = 0.40 and the bleeding index *p* = 0.24. A comparison of the pre- and post-treatment plaque and bleeding values of the two test groups and the control group showed a statistically significant result for *p* < 0.01.

## 4. Discussion

Traditionally, the beneficial properties of extra-virgin olive oil have been attributed to its high content of monounsaturated fatty acids, which represent up to 80% of its total lipid composition. Recent evidence has shown that the minor components of extra-virgin olive oil, such as phenolic compounds and other compounds with antioxidant activity, determine an increase in the healthy characteristics of the oil itself.

In the scientific literature, there are few studies on the use of extra-virgin olive oil as an adjuvant for gingival inflammation. In the previous chapter, some scientific articles described the state of the art in which natural mouthwashes are used. The data obtained in the literature can be compared with the results obtained from the experimental studies conducted [20,21]. These studies showed that the two groups that used mouthwash with olive oil had a statistically significant result. The groups subjected to the treatment had a very positive change in the bleeding and plaque indices, in terms of percentage improvement, compared to those of the group subjected to non-surgical mechanical therapy. As in a study carried out in 2003 by Pretty IA et al. [20] on the use of an olive oil-based toothpaste and that of a study carried out in 2020 by Nardi G.M. et al. [21], on the use of mouthwash composed of ozonized olive oil, treatment with olive tree-based products is an excellent adjuvant for maintaining oral hygiene at home.

The encouraging results of the current study mirror the ones in the literature [22]; therefore, further research regarding this traditional, affordable, and beneficial technique should be encouraged and carried out. When performed properly, it can be safely used as a coadjuvant for maintaining good oral health and hygiene if it is always combined with tooth brushing and flossing.

Remember that while some individuals report improvements in their oral health after oil pulling, the scientific evidence is limited. The American Dental Association (ADA) has stated that there is insufficient evidence to support the use of oil pulling as a substitute for traditional oral hygiene practices in managing gum disease. It is always best to consult with a dental professional for a personalized treatment plan for gingivitis and to maintain good oral hygiene practices as recommended by your dentist [23].

A further aspect to consider in the use of oil pulling is the environmental impact. The environmental impact of using oil pulling as a treatment for gingivitis is generally considered to be lower compared to some other dental practices that involve the use of commercial oral care products.

## 5. Conclusions

The oil pulling treatment with extra-virgin olive oil mouthwash was effective in the treatment of plaque-induced gingivitis; it did not show any negative effects or disadvantages except for the longer duration of the procedure (5 min) compared to other mouthwashes (2 min). This type of oil pulling treatment with extra-virgin olive oil has proven to be a good adjuvant for maintaining home oral hygiene thanks to its ability to reduce the adhesion and formation of bacterial plaque. While there were improvements noted in this study, the exact mechanism of this treatment remains to be elucidated. Because of this, it is necessary to carry out further studies with a different sample of patients than those used and a comparison with other products to verify and demonstrate the antibacterial and anti-inflammatory effects of the components of this natural product.

## Figures and Tables

**Table 1:** Percentages history sheet: test group and control group.

	EVO	Fruity	Control
Sex: *n* (*%*)			
Women	14 (10.5%)	19 (14.3%)	11 (8.2)
Men	8 (6%)	9 (6.75%)	14 (10.5%)
Medication: *n* (*%*)			
No medication	22 (16.5%)	26 (19.5%)	22 (16.5%)
Anticoagulants	1 (∼1%)	1 (∼1%)	0
Other	0	1 (∼1%)	3 (2.25%)
Smoke *n* (*%*)	8 (6%)	6 (4.5%)	9 (6.75%)
Toothbrush *n* (*%*)			
Electric	15 (11.25%)	4 (3%)	1 (∼1%)
Manual	7 (5.25%)	23 (17.25%)	24 (18%)
Electric and manual	0	1 (∼1%)	0

**Table 2:** Comparison of EVO oil group mean values of plaque index and bleeding index at t0 and t2. Dev.St: standard deviation, standard error of the mean; t0: before treatment; t2: 30 days after EVO oil treatment.

	N	Mean	Standard Deviation	Standard Mean Error
Plaque T0	25	0.96	0.200	0.040
Plaque T2	25	0.48	0.510	0.102
Bleeding T0	25	0.96	0.200	0.040
Bleeding T2	25	0.32	0.476	0.095

**Table 3:** Comparison of fruity oil group mean values of plaque index and bleeding index at t0 and t2. Dev. St: standard deviation, standard error of the mean; t0: before treatment; t2: 30 days after treatment with fruity oil.

	N	Mean	Standard Deviation	Standard Mean Error
Plaque T0	25	0.92	0.277	0.055
Plaque T2	25	0.56	0.507	0.101
Bleeding T0	25	0.84	0.374	0.075
Bleeding T2	25	0.44	0.101	0.101

**Table 4:** (a). Comparison of control group mean values of plaque index and bleeding index at t0 and t2. Dev.St: standard deviation, standard error of the mean; t0: before treatment; t2: 30 days after treatment without any mouthwash. (b). Comparison of mean values of the three groups of plaque index (IP) and bleeding index (IS) at t0 and t2 * A *p* value (significance level) < 0.05 is considered statistically significant (Student’s *t*-test). IP: plaque index; IS: bleeding index; Dev.St: standard deviation; t0: before treatment; t2: 30 days after treatment.

(a)				
	N	Mean	Standard Deviation	Standard Mean Error
Plaque T0	25	0.88	0.332	0.066
Plaque T2	25	0.96	0.200	0.040
Bleeding T0	25	0.92	0.277	0.055
Bleeding T2	25	0.84	0.374	0.075
**(b)**				
**Group**		**Mean**	**Standard Deviation**	*p* (Level of Significance) *
IP T0		0.96	0.200	
EVO oil group		0.92	0.277	0.008770647
Fruity group Control group		0.88	0.332	
IS T0		0.96	0.200	
EVO oil group		0.84	0.374	0.01829958
Fruity group Control group		0.93	0.277	
IP T2		0.48	0.510	
EVO oil group		0.56	0.507	0.40707794
Fruity group Control group		0.96	0.200	
IS T2		0.96	0.476	
EVO oil group		0.44	0.507	0.24496896
Fruity group Control group		0.84	0.374	

**Table 5:** Comparison of pre- and post-treatment IP and IS of the EVO oil group, fruity oil group, and control group. * A *p* value (significance level) < 0.05 is considered statistically significant (Student’s *t*-test). IP: plaque index, IS: bleeding index.

Group	Mean		*p* (Level of Significance) *
IP/IS	IP	IS	
EVO oil group T0	0.96	0.96	0.420833152
EVO oil group T2	0.48	0.32	
IP/IS	IP	IS	
Fruity group T0	0.92	0.84	0.125665916
Fruity group T2	0.56	0.44	
IP/IS	IP	IS	
Control group T0	0.88	0.92	0.704832765
Control group T2	0.96	0.84	

## Data Availability

Data are available upon request to the corresponding author.
